# Notice of a Suddenly Fatal Disease, at Stamford, Kentucky

**Published:** 1833

**Authors:** 


Art. VII. Notice of a suddenly fatal disease, at Stanford, Kentucky, *in a letter to the Editor from L. G. Mullens, dated Nov. 20th*, 1833.

"Permit me to make some statements respecting a disease, that is now raging with great mortality in this vicinity. Almost every case has terminated fatally. I call it congestive fever, but there is great difference in opinion among the physicians here, respecting its nature and treatment. I will give you the symptoms, and our treatment, so that you may form an opinion, and I am in hopes throw some light on the subject.' The first case was that of Mr. H. H., aged about forty-five, stout, and of a robust constitution and sanguineous temperament. Previous to his attack he worked hard all day, and ate a hearty supper. About midnight, he was taken with cold chills, soon followed with a violent pain in his head, redness of the eyes, delirium, attended with pain in his side and breast, great restlessness, a quick and hard, but not full pulse, involuntary sighing, sensation of weight about the pit of the stomach, and great nausea. His countenance soon acquired a look of extreme agitation, the skin was dry and hot, and the centre of the tongue white, but moist. Evidences of still greater irregularity of excitement soon developed themselves; the pulse now became small and hurried, the heat sharp and concentrated about the precordia, but lower than natural, in the wrists, ankles, forehead and lobes of the ears. In the beginning of the attack, Dr. Huff and myself, who were called on, bled him freely, under which operation his pulse became fuller and less frequent, but he was still affected with violent delirium, and could hardly be held in the bed. After the blood-letting, we gave him drastic purgatives, but found great difficulty in promoting an operation on the bowels, purgatives together with injections being exhibited throughout the night without much effect. The first discharges were almost natural, but after a few evacuations, they became dark and very offensive. In the morning, the blood broke out from the arm. I let him bleed again freely, after which his pulse became small and his face bloated, but his skin, in spots, was dry and hot, and he remained entirely out of his senses; we then applied blistering plasters on the head, side, breast and extremities. In the morning of the second day of his attack, we again visited him, and found him somewhat more composed, but weak and considerably stupid. The cutaneous excitement still irregular, the surface being hot in some parts, and cold in others, and partially moist; the delirium continuing without intermission; his bowels were kept open by active purgatives. Towards night he became more composed, and got more in his senses; his skin also became generally moist, and he began to complain of his blistering plasters; but his eyes still continued red, and his countenance wild at night. We left him with medicines to keep his bowels open, and spirits of nitre to maintain moisture on the surface. In the morning we found him decidedly better; more in his senses, his pulse soft and regular, no particular pain, and his skin moist. We continued the antiphlogistic treatment. About the following midnight, the medicines failed to operate on his bowels, and he became so delirious and raving, that he could hardly be held in the bed; but by a more active course of purgatives, soon got his bowels into action, when he grew much quieter. We continued the same method, and under it he improved rapidly for several days; but still complained of great soreness of his head. His appetite at length became craving, and his system began to strengthen. But when all his symptoms were flattering, he ate grapes and persimmons, which threw him back with all the symptoms he first had; and after experiencing great torture, with delirium and a strong tendency to convulsions, he expired in a few hours.

Mr. A. H., the brother, died on the same night, about two hours from the time he was confined to bed. He was much more convulsed, and his case was attended with vomiting.

The following morning we left the family all in good health. About twelve o'clock, Mrs. H., the widow of Mr. H. H., was taken with the same disease, and expired about ten o'clock at night.

Mr. J. C., a neighbor, who sat up with the family, was taken, a day or two afterwards, and the disease soon terminated fatally.

Other cases have occurred in the same neighborhood. We have been successful in the treatment of very few, as the disease terminates in death before the physician has much time to use efficient remedies. Indeed, as to the symptoms and result, the cases above cited may be received, so far as my observation has gone, as tolerably fair examples of the whole.

I and my medical friends would be much gratified to receive your opinion and advice relative to the nature and treatment of this malady, or that of any other physician, should you see proper to publish this hasty letter in the Western Journal."

### Editorial Observations.

It cannot be doubted, we think, that in the cases narrated by Dr. Mullens, the brain was deeply implicated. But when a patient dies with an affection of that or any other organ, after an illness of a few hours, or a day, it certainly is not inflammation that destroys him. The time is too short for that variety of morbid action to establish itself, much less to run on to a fatal termination. Doubtless there was a great accumulation of blood in the brain, but the morbid action was epileptic, rather than apoplectic or arachnitic. We should presume that the cold dash or current on the head, after bloodletting, would have been serviceable.

As to the remote cause of this malady, we may suppose that it was nothing more nor less, than that which produced the late Epidemic Cholera, modified, however, by some other cause, of which we are equally ignorant. Great epidemics have often been preceded, attended and followed, by anomalous and fatal cases of disease, which the profession have, generally, and we think correctly, referred to the unknown noxious agents that produce them.

We hope that some of our correspondents will elucidate this matter. We shall be happy to publish their speculations; and may here take occasion to say, that we think our Journal might be made a useful vehicle of communication among the physicians of the West; who could reciprocally afford advice to each other, on the new forms of disease which are perpetually arising. It would at all times gratify us to facilitate such a correspondence.

